# Value of quantitative ultrasound and bioelectrical impedance analysis in detecting low bone mineral density in hemodialysis

**DOI:** 10.1080/0886022X.2021.1959347

**Published:** 2021-08-09

**Authors:** Ting Xiang, Li Zhou, Ping Fu, Xue-Ping Yan, Xiao-Qing Zeng

**Affiliations:** a Division of Nephrology, Kidney Research Institute, West China Hospital of Sichuan University, Chengdu, Sichuan, China; b Nutrition Department, West China Hospital of Sichuan University, Chengdu, Sichuan, China

**Keywords:** Low bone mineral density, quantitative ultrasound, bioelectrical impedance analysis, osteoporosis, hemodialysis

## Abstract

**Introduction:**

Patients on maintenance hemodialysis (MHD) are highly predisposed to low bone mineral density (BMD). This study aims to assess the value of quantitative ultrasound (QUS), bioelectrical impedance analysis (BIA), and their combination in detecting high-risk patients for low BMD in MHD.

**Methods:**

Patients’ BMD of the total hip, femoral neck, and lumbar spine were measured using dual-energy X-ray absorptiometry (DXA). Bone mineral content (BMC) was assessed using BIA. Calcaneal BMD was measured using QUS. Patients with a T-score of ≤-2.5 were recorded as ‘low BMD.’

**Results:**

Overall, 93 subjects (62.37% female; mean age, 60.8 ± 16.2 years) were included in this cross-sectional study; approximately 36.56% met the ‘low BMD’ criteria. QUS-T score predicted low BMD with an area under the curve (AUC) value of 0.738, sensitivity of 70.59%, and specificity of 76.27%. The AUC for low BMD diagnosis using the BMC index (BMCI) measured through BIA was 0.679 (sensitivity, 91.18%; specificity, 38.98%). On the other hand, the combination of QUS-T score and BMCI yielded a higher AUC value of 0.762 with an improved specificity of 88.14%. Compared with the QUS and BIA alone, the net reclassification improvement (NRI) of the combination model increased by 47.16% (*p* = 0.022) and 78.36% (*p* < 0.0001), respectively. Integrated discrimination improvement (IDI) increased by 5.25% (*p* = 0.043) and 9.99% (*p* = 0.003), respectively. QUS-T score and BMCI were related to BMD independently assessed by DXA.

**Conclusion:**

The combination of QUS and BIA is effective in screening for low BMD among MHD patients.

## Introduction

Patients with end-stage renal disease (ESRD) are predisposed to various metabolic bone disorders, with a fourfold higher risk of hip fracture than did the general population [1]. Osteoporosis (OP) is a common disease in patients with ERSD, causing falls and fracture; it is characterized by decreased bone mineral density (BMD) and the destruction of bone microarchitecture [2]. For patients with ESRD and low-trauma fractures, except for chronic kidney disease mineral and bone disorder (CKD-MBD), clinicians should check for coexisting OP. The bone status of patients with advanced CKD is compromised in a complex way, as shown in bone biopsy, which is invasive and rarely clinically available [3]. Therefore, noninvasive techniques such as dual-energy X-ray absorptiometry (DXA) are increasingly used to evaluate the bone status of patients with advanced CKD [4]. Several prospective studies confirmed that reduced BMD was an independent risk factor for fragility fractures even among patients with CKD stage 3–5 D [5,6]. As recommended by the 2017 Kidney Disease: Improving Global Outcomes Guideline, BMD measurement is suitable for patients with CKD stage 3a–5D who have risk factors for OP [7]. Currently, the ‘gold standard’ for BMD assessment is based on DXA [8]. However, DXA is costly and requires highly trained operators, meaning it could only be available in certain big medical facilities. Thus, there is an urgent need to develop simple and more available methods of low bone mass screening for high-risk individuals in local hospitals. With its ability to detect the bone quantity and bone structure/elasticity [9,10], quantitative ultrasound (QUS) is being used worldwide for OP screening because of its low cost and portability. However, not many studies have assessed the value of QUS in detecting OP among patients on hemodialysis [11,12], and these studies did not mention the optimal QUS-T score of the diagnosis of OP for patients on maintenance hemodialysis (MHD). In addition, bioelectrical impedance analysis (BIA) provides body composition measurements with fast processing, is radiation-free, and is available even in community hemodialysis [13]. BIA is also used in estimating bone mineral content (BMC), which is associated with broadband ultrasound attenuation measured using QUS in the general population [14]. These studies suggested that BIA could be a useful method of detecting OP; however, this method has not been studied in patients on hemodialysis and has not been recommended for diagnostic purposes.

This study aims to investigate the validity of QUS and BIA in screening high-risk individuals with low BMD and explore the optimal cutoff values in patients on hemodialysis, which contributes to finding a simple and economical screening method for low BMD.

## Materials and methods

### Subjects

This cross-sectional study included a cohort of patients with ESRD under MHD in the West China Hospital, Sichuan University. The inclusion criteria were (1) being under MHD for 4 h thrice weekly for more than 3 months and (2) agreement to participate in this study. We excluded those who underwent parathyroidectomy; those who used bisphosphonates in the last 6 months; those for whom BIA could not be performed (such as those patients who underwent pacemaker installation, artificial joint replacement, and amputation surgery and patients with severe peripheral angiopathy); those who received kidney transplants; and those with nonrenal diseases, such as cancer and tuberculosis, that seriously affect bone metabolism.

### Data collection and measurements

Data on the participants’ clinical characteristics and maintenance medication were collected. Biochemical parameters were detected within one month of enrollment. QUS (Hologic Sahara, USA) was performed to measure the calcaneal BMD of the right heel and determine the T-score. BMC was assessed after hemodialysis using BIA (InbodyS720, Biospace, Seoul, South Korea). The BMC index (BMCI) was calculated using the following formula: BMC (kg)/height^2^ (m^2^). The BMD of the total hip, femoral neck, and lumbar spine (L1–L4) were evaluated using DXA (GE Lunar, ME + 212243, USA) and BMD (g/cm^2^). T-scores were recorded. Patients on MHD may have osteoporotic bone and bone mineral disorders related to CKD, making the diagnosis of OP relatively trickier [15]. Therefore, patients with a T-score of ≤ −2.5 were recorded as ‘low BMD’ in our study.

### Statistical analyses

Data were analyzed using SPSS version 21.0 (IBM, Armonk, NY), MedCalc version 17.6 (MedCalc Software BVBA, Ostend, Belgium), and R version 3.0.2 (The R Foundation for Statistical Computing). The Kolmogorov–Smirnov statistic was used to test variable normality. The chi-square test was used to analyze the difference between groups for qualitative variables. The *t-*test or Mann–Whitney U test was used for quantitative variables according to the distribution and equality of variance. The ability of BIA and QUS to identify low BMD was assessed using the receiver operating characteristic (ROC) curve analysis. The continuous versions of the net reclassification improvement (NRI) and integrated discrimination improvement (IDI) were used to compare the diagnostic accuracy and discrimination of the combination of BIA and QUS with the BIA and QUS alone. Binary logistic regression was used for the disease prediction equation. Pearson’s linear analysis was used to test the correlation between QUS-T score, BMCI, and DXA-T score. Multiple linear regression analysis (stepwise method) was used to identify the parameters affecting BMD. The variance inflation factor (VIF) was used to evaluate the multicollinearity of regression models. A VIF above 2.5 indicates that the model has multicollinearity. *p* < 0.05 was considered statistically significant.

## Results

### Patient demographics

A total of 93 subjects were included in this study. The mean age of the study participants was 60.8 ± 16.2 years (range, 21–88), and 58 (62.36%) were female. Table 1 shows the demographic data. A total of 34 (36.56%) patients met the criteria for low BMD, and females were more prone to bone mass loss than did males (*p* < 0.001). The BMCI and QUS-T score were significantly reduced in patients with low BMD compared with controls (1.03 ± 0.11 vs. 1.12 ± 0.15, *p* = 0.002; −2.31 ± 1.02 vs. −1.57 ± 0.98, *p* = 0.001). The C-reactive protein (CRP), Kt/v, and fracture rates were higher in patients with low BMD than in those without low BMD.

**Table 1.** Characteristics of the participants in the study (*n* = 93).

	Low BMD (*n* = 34)	Non-low BMD (*n* = 59)	*p*
Age (years)	64.62 ± 13.99	58.59 ± 17.04	0.084
Sex			<0.001
Male（*n* = 35）	4 (11.43%)	31 (88.57%)	
Female（*n* = 58）	30 (51.72%)	28 (48.28%)	
Dialysis vintage (years)	4.5 (3, 8.25)	5 (3.5, 8)	0.269
BMI (kg/m^2^)	22.03 ± 3.47	23.12 ± 3.24	0.134
BMD (g/cm^2^)			
Total hip	0.66 ± 0.1	0.87 ± 0.13	<0.001
Femoral neck	0.62 ± 0.87	0.81 ± 0.12	<0.001
Lumbar spine	0.83 ± 0.15	1.11 ± 0.23	<0.001
BMCI (kg/m^2^)	1.03 ± 0.11	1.12 ± 0.15	0.002
QUS-T score	−2.31 ± 1.02	−1.57 ± 0.98	0.001
^a^Ca (mmol/L)	2.36 ± 0.22	2.29 ± 0.25	0.222
P (mmol/L)	1.87 ± 0.49	1.84 ± 0.47	0.762
PTH (pmol/L)	20.79 (12.24, 38.29)	27.07 (13.31, 50.6)	0.192
ALP (IU/L)	77 (63, 106)	85 (67, 111)	0.382
25(OH)D (nmol/L)	46.32 ± 18.93	51.63 ± 20.98	0.251
β2-MG (mg/L)	42.49 ± 13.78	40.04 ± 10.21	0.328
CRP (mg/L)	5.65 (3.32, 9.81)	3.22 (2.28, 6.37)	0.046
Albumin (g/L)	40.89 ± 3.73	41.97 ± 4.25	0.227
Kt/v	1.59 ± 0.31	1.44 ± 0.36	0.048
^b^Vitamin D analogs, *n*(%)	19 (55.88%)	40 (67.8%)	0.251
^b^Phosphorus binders, *n*(%)	10 (29.41%)	20 (33.9%)	0.656
^c^Glucocorticoids therapy, *n*(%)	14 (41.17%)	16 (27.12%)	0.163
Fracture history, *n*(%)	12 (35.3%)	6 (10.17%)	0.003
Diabetes, *n*(%)	9 (26.47%)	22 (37.29%)	0.287
Coronary artery disease, *n*(%)	4 (11.76%)	7 (11.86%)	0.989

BMI: body mass index; BMD: bone mineral density; BMCI: bone mineral content index; QUS-T: quantitative ultrasound T score; Ca: calcium; P: phosphate; PTH: parathyroid hormone; ALP: alkaline phosphatase; 25(OH)D: 25-hydroxyvitamin D; β2-MG: β2-microglobulin; CRP: C-reactive protein.

^a^Ca was calculated by the following formula: [40–serum albumin(g/L)] × 0.02 +serum calcium (mmol/L).

^b^Vitamin D analogs and phosphorus binders were defined as receiving the drugs for more than 2 month before enrollment.

^c^Glucocorticoids therapy was recorded if the patient is currently taking oral glucocorticoids or has taken oral glucocorticoids for more than 3 months, and the daily dose of 5 mg or more.

### Diagnostic performance of QUS-T and BMCI for predicting low BMD

**Figure 1.**
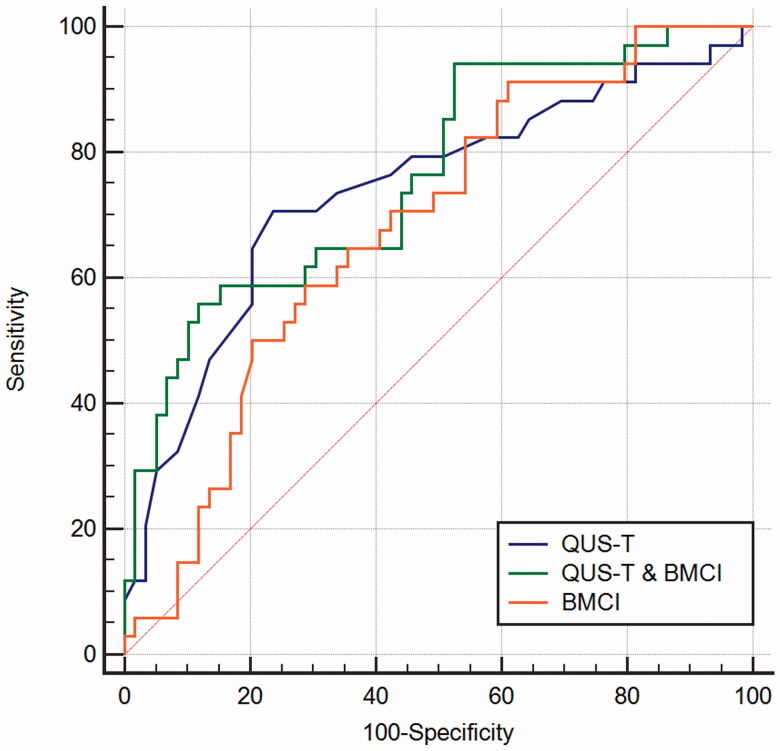
The receiver operating characteristic curve of the BMCI, QUS-T, and their combination in predicting MHD with low BMD. QUS-T: quantitative ultrasound T score; BMCI: bone mineral content index; QUS-T and BMCI: the combination of the BMCI and QUS-T score; MHD: maintenance hemodialysis; BMD: bone mineral density

The ROC curve was used to assess the validity of the QUS-T score and BMCI in detecting low BMD (Figure 1, Table 2). The QUS-T score indicated low BMD, with an area under the curve (AUC) of 0.738 (95% confidence interval (CI), 0.637–0.824). The optimal cutoff value for low BMD was −2.3. The AUC for the diagnosis of low BMD by the BMCI was 0.679 (95% CI, 0.574–0.772), with the best cutoff value of 1.16 kg/m^2^. The sensitivity and specificity of the QUS-T score were 70.59% and 76.27%, respectively, and that for BMCI were 91.18% and 38.98%, respectively. We calculated the incidence probability (*p*) of low BMD based on the BMCI and QUS-T score using the logistics regression analysis. *p* = 2.851–0.82 QUS-T score −4.7 BMCI. We then performed the ROC analysis of P to identify low BMD. The combination of the QUS-T score and BMCI yielded a higher AUC of 0.762 (0.663–0.844), with a specificity of 88.14%, which was optimized compared with the QUS-T score or BMCI alone.

**Table 2.** Discriminatory performance of QUS-T score and BMCI for predicting low BMD.

	AUC (95% CI)	*p*	Cutoff	SE (%)	SP (%)	PPV (%)	NPV (%)
QUS-T	0.738 (0.637–0.824)	<0.001	−2.3	70.59	76.27	63.2	81.8
BMCI	0.679 (0.574–0.772)	0.004	1.16	91.18	38.98	46.3	88.5
QUS-T & BMCI	0.762 (0.663–0.844)	<0.001	–	55.88	88.14	73.1	77.6

QUS: quantitative ultrasound; BMCI: bone mineral content index; QUS-T & BMCI: combination of BMCI and QUS-T score; AUC: area under the curve; CI: 95% exact confidence intervals; SE: sensitivity; SP: specificity; PPV: positive predictive value; NPV: negative predictive value.

### Comparison of the diagnostic performance of the QUS-T and BMCI and QUS-T and BMCI

As shown in Table 3, the AUC of the combination of the BMCI and QUS-T was 0.762, which was larger than that of the BMCI (0.679, *p* = 0.129) and QUS-T (0.738, *p* = 0.545). However, there was no statistical significance for the two outcomes. According to the NRI analysis, the combination of the BMCI and QUS-T significantly improved the accuracy of the identification of low BMD (QUS alone: NRI, 47.16%; *p* = 0.022; BMCI alone: NRI, 78.36%; *p* < 0.0001). IDI analysis showed that the discrimination of the combination of the BMCI and QUS-T was higher than that of the QUS alone (IDI, 5.25%; *p* = 0.043) and the BMCI alone (IDI, 9.99%; *p* = 0.003).

**Table 3.** Comparison of the diagnostic performance of the QUS-T & BMCI and QUS-T and BMCI.

Model	AUC (95%CI)	NRI,% (95%CI)	IDI,% (95%CI)
QUS-T	0.738		
QUS-T&BMCI	0.762	47.16 (6.77 − 87.55)	5.25 (0.17 − 10.33)
p	0.545	0.022	0.043
BMCI	0.679		
QUS-T&BMCI	0.762	78.36 (40.65 − 116.08)	9.99 (3.4 − 16.59)
p	0.129	<0.0001	0.003

QUS: quantitative ultrasound; BMCI: bone mineral content index; QUS-T & BMCI: combination of BMCI and QUS-T score; AUC: area under the curve; CI: 95% exact confidence intervals; NRI: net reclassification improvement; IDI: integrated discrimination improvement.

### Correlation between QUS-T, BMCI, and BMD

Table 4 shows the Pearson’s correlation between the QUS-T score, BMCI, and BMD measured using DXA. The QUS-T score was significantly correlated with the BMD of the total hip, femoral neck, and lumbar spine. The BMCI was also correlated with the BMD of all sites, while the correlation coefficients were poorer than the QUS-T score (scatterplots were shown as Supplemental Figures). The multiple linear regression results showed that the QUS-T score and BMCI remain significantly correlated with the BMD of the total hip and femoral neck after adjusting for age, sex, fracture history, coronary artery disease, diabetes, weight, vitamin D analogs and phosphorus binder usage, glucocorticoid therapy, dialysis vintage, calcium, phosphate, alkaline phosphatase, 25-hydroxyvitamin D, parathyroid hormone, CRP, and potential contenders, while correlation with the lumbar spine was no longer visible (Table 5).

**Table 4.** Pearson's correlation between QUS-T score, BMCI and bone mineral density (BMD) in subjects.

	Total hip		Femoral neck		Lumbar spine	
	*r*	*p*	*r*	*p*	*r*	*p*
QUS-T score	0.598	*p* < 0.001	0.526	*p* < 0.001	0.407	*p* < 0.001
BMCI	0.418	*p* < 0.001	0.383	*p* < 0.001	0.212	0.046

QUS: quantitative ultrasound; BMCI: bone mineral content index.

**Table 5.** Multiple regression models for QUS-T score, BMCI associated with BMD in total hip, femoral neck and lumbar spine.

	BMCI					QUS-T				
	*β*	95%CI	SE (*β*)	*t*	*p*	*β*	95%CI	SE (*β*)	*t*	*p*
Model 1										
Total hip	0.46	0.25–0.67	0.106	4.326	<0.001	0.087	0.063–0.112	0.012	7.075	<0.001
Femoral neck	0.377	0.188–0.567	0.095	3.939	<0.001	0.07	0.047–0.094	0.012	5.871	<0.001
Lumbar spine	0.356	0.006–0.705	0.176	2.024	0.046	0.092	0.048–0.136	0.022	4.162	<0.001
Model 2										
Total hip	0.348	0.147–0.549	0.101	3.445	0.001	0.072	0.047–0.097	0.013	5.627	<0.001
Femoral neck	0.286	0.098–0.474	0.095	3.027	0.003	0.064	0.04–0.088	0.012	5.26	<0.001
Lumbar spine	0.032	–	–	0.312	0.756	0.063	0.02–0.107	0.022	2.883	0.005
Model 3										
Total hip	0.308	0.096–0.52	0.107	2.888	0.005	0.066	0.04–0.092	0.013	5.015	<0.001
Femoral neck	0.245	0.046–0.443	0.1	2.457	0.016	0.065	0.041–0.089	0.012	5.398	<0.001
Lumbar spine	–0.057	–	–	–0.507	0.614	0.195	–	–	1.831	0.071

QUS: quantitative ultrasound; BMCI: bone mineral content index.

Model 1: Unadjusted.

Model 2: Adjusting for age, sex, fracture history, coronary artery disease, diabetes, weight.

Model 3: Model 2 and Vitamin D analogs and phosphorus binders usage, glucocorticoids therapy, dialysis vintage, calcium, phosphate, alkaline phosphatase, 25-hydroxyvitamin D, parathyroid hormone, CRP.

## Discussion

Our results suggested that the BMCI and QUS-T score were useful in realizing that hemodialysis with low BMD and QUS might have a better testing effect than did BIA. However, the BMCI had a better sensitivity of 91.18% and a negative predictive value (NPV) of 88.5%, indicating that BIA can sensitively screen out high-risk individuals to be referred for DXA testing. The higher NPV meant a lower false-negative rate, which can accurately exclude low-risk subjects of low BMD and reduce the rate of unnecessary DXA tests. The combination of the QUS and BIA yielded a higher effect and had a better specificity of 88.14% and a positive predictive value (PPV) of 73.1%, suggesting greater abilities to screen out the diagnosed patients. QUS and BIA could be alternative methods of detecting low BMD in areas where DXA measurement is unavailable.

Previous studies showed a moderate association between calcaneal parameters and DXA (*r* = 0.32–0.53, *p* < 0.05), which was similar to our results, and a higher AUC (0.80) of QUS in the diagnosis of OP than that of our study [11,12]. The best QUS cutoffs in detecting OP were only observed in the general population [16]. The difference between the general population and patients on dialysis is that the latter lost more cortical bone than trabecular bone [17]. The hip is made up of mostly cortical bone, while the calcaneus is trabecular bone, and QUS measures calcaneus. Therefore, theoretically, the QUS-T score cutoff in diagnosing OP among patients on dialysis should be different from that in the general population. Finding the optimal cutoff value of the QUS-T score among patients on dialysis is of great importance. Previous studies have shown a correlation between QUS measurements and DXA results in hemodialysis [11,18]. However, to our knowledge, our study is the first to put forward the best cutoff value of the QUS-T score in predicting OP in hemodialysis. Because of the unique characteristics of bone metabolism, more large-scale studies are still needed to find the optimal cutoff value of QUS-T score and BMCI in MHD.

BIA is a noninvasive, reliable method of assessing physical status and dry weight, which has been recommended for patients with MHD [19]. BIA can also be used to estimate the BMC, which might be useful for bone density evaluation and monitoring [20]. A recent study reported that the BIA value was related to calcium, phosphorus, and parathyroid hormone (PTH) in patients on MHD, which might be of significant application value for the assessment and prevention of CKD-MBD [21]. Our study found that the BMCI was significantly associated with the BMD measured by DXA, and we reported the BMCI value in detecting low BMD in hemodialysis with the best cutoff value of 1.16 kg/m^2^. BIA is expected to be a new tool for evaluating bones in patients on dialysis. In addition, the combination of QUS and BIA improved the effectiveness of screening for low bone mass with a higher AUC, NRI, and IDI values and a better specificity, suggesting that the combination of these two simple methods might also be a good choice.

Strictly speaking, the definitions of low BMD adopted in this study are based on the World Health Organization criteria, which is beyond the age of 50 years for men and postmenopausal women. For this study, we applied this cutoff value for all subjects. However, our results would be impacted by the cutoff value of the DXA-T score. To further demonstrate that QUS and BIA can be used for BMD assessment, we performed a correlation analysis, whose results showed a significant correlation between the QUS-T score and the BMCI and DXA-T score in any site. After adjusting for potential confounders, the QUS-T score and BMCI still maintained the correlation with the BMD of the total hip and femoral neck. However, the correlation for the lumbar spine was no longer noted. The DXA relies on the relative absorption of X-ray beams. In addition, dialysis affects arterial calcification. X-rays may be absorbed by the calcified aorta instead of the spine, which might lead to elevated lumbar BMD measurements in the anterior–posterior (AP) position. It was reported that the differences in the AP spine BMD and femoral neck BMD had a positive correlation with abdominal aortic calcification [22]. In addition, the lumbar BMD measured using CT scans significantly correlated with lumbar T-scores from the lateral DXA, but not with those from AP DXA [23]. Therefore, the lumbar spine is not an appropriate site of BMD measurement for these patients. However, lateral DXA is capable of avoiding the overestimation of BMD with aortic calcification, making it a reliable method for measuring the lumbar BMD.

Our study has certain limitations. First, it was a single-center study with a small sample size; thus, the study sample was not quite representative of the actual population. Second, the BIA depends on body hydration, which often fluctuates in patients on MHD. Therefore, it is better to perform measurements after dialysis, while it may still have an influence on the measurement. However, to the best of our knowledge, our study is the first to put forward the combination of QUS and BIA for low BMD screening in patients on MHD. The findings of our study presented a new perspective of the tools used in the screening of low BMD in patients on hemodialysis. We also pointed out the necessity of finding a cutoff value of QUS in diagnosing low BMD for patients on MHD.

## Conclusion

Evaluating the bone status in patients on MHD via bone biopsy is difficult. DXA scans cannot distinguish true low bone mass from osteomalacia because of impaired mineralization. However, DXA can noninvasively identify patients with low BMD for further evaluation. Individualized anti-OP treatment based on bone metabolism indicators and BMD could reduce the incidence of fractures. Early screening of patients on MHD at high-risk of low BMD is necessary. QUS is widely used for screening OP in the community. BIA is also available to measure the nutritional status and dry weight of patients in some dialysis institutions. The simple and noninvasive methods of QUS and BIA may constitute the first step in screening for low BMD, especially in medical facilities where DXA is not accessible. Subjects with a high-risk of low bone mass indicated that patients screened using this method should be referred to undergo further DXA tests for definitive diagnosis.

## Supplementary Material

Supplemental Material

## References

[1] Alem AM, Sherrard DJ, Gillen DL, et al. Increased risk of hip fracture among patients with end-stage renal disease. Kidney Int. 2000;58(1):396–399. 10886587 10.1046/j.1523-1755.2000.00178.x

[2] Brunerova L, Ronova P, Veresova J, et al. Osteoporosis and impaired trabecular bone score in hemodialysis patients. Kidney Blood Press. 2016;41(3):345–354. 10.1159/000443439 27333273

[3] Malluche H, Faugere MC. Renal Bone disease 1990: an unmet challenge for the nephrologist. Kidney Int. 1990;38(2):193–211. 2205749 10.1038/ki.1990.187

[4] Kuo CW, Ho SY, Chang TH, et al. Quantitative ultrasound of the calcaneus in hemodialysis patients. Ultrasound Med Biol. 2010;36(4):589–594. 20211518 10.1016/j.ultrasmedbio.2009.12.003

[5] Naylor KL, Garg AX, Zou G, et al. Comparison of fracture risk prediction among individuals with reduced and normal kidney function. CJASN. 2015;10(4):646–653. 25655423 10.2215/CJN.06040614 PMC4386249

[6] West SL, Lok CE, Langsetmo L, et al. Bone mineral density predicts fractures in chronic kidney disease. J Bone Miner Res. 2015;30(5):913–919. 25400209 10.1002/jbmr.2406

[7] Kidney Disease: Improving Global Outcomes (KDIGO) CKD-MBD Update Work Group. KDIGO 2017 Clinical practice guideline update for the diagnosis, evaluation, prevention, and treatment of chronic kidney disease–mineral and bone disorder (CKD-MBD). Kidney Int Suppl. 2017;7:1–59. 10.1016/j.kisu.2017.04.001 PMC6340919 30675420

[8] World Health Organization. Assessment of fracture risk and its application to screening for postmenopausal osteoporosis. WHO technical report series. . 1994;843:1–129. 7941614

[9] Njeh CF, Fuerst T, Diessel E, et al. Is quantitative ultrasound dependent on bone structure? A Reflection. Osteoporos Int. 2001;12(1):1–15. 11305077 10.1007/PL00020939

[10] Bouxsein ML, Radloff SE. Quantitative ultrasound of the calcaneus reflects the mechanical properties of calcaneal trabecular bone. J Bone Miner Res. 1997;12(5):839–846. 9144351 10.1359/jbmr.1997.12.5.839

[11] Arici M, Erturk H, Altun B, et al. Bone mineral density in haemodialysis patients: A comparative study of dual-energy X-ray absorptiometry and quantitative ultrasound. Nephrol Dial Transplant. 2000;15(11):1847–1851. 11071976 10.1093/ndt/15.11.1847

[12] Taal MW, Cassidy MJ, Pearson D, et al. Usefulness of quantitative heel ultrasound compared with dual-energy X-ray absorptiometry in determining bone mineral density in chronic haemodialysis patients. Nephrol Dial Transplant. 1999;14(8):1917–1921. 10462271 10.1093/ndt/14.8.1917

[13] Rikkonen T, Sirola J, Salovaara K, et al. Muscle strength and body composition are clinical indicators of osteoporosis. Calcif Tissue Int. 2012;91(2):131–138. 22733383 10.1007/s00223-012-9618-1

[14] Feher P, Annar D, Zsakai A, et al. The body composition analysis as a complementary tool in the screening of bone structural abnormalities. anthranz. 2020;77(2):161–171. 10.1127/anthranz/2020/1136 32142092

[15] Hallock A. Osteoporosis in patients with CKD: a diagnostic dilemma. Nephrol Nurs J. 2017;44(1):13–17. 29237104

[16] Flöter M, Bittar CK, Zabeu JL, et al. Review of comparative studies between bone densitometry and quantitative ultrasound of the calcaneus in osteoporosis. Acta Reumatol Port. 2011;36(4):327–335. 22472924

[17] Duan Y, De Luca V, Seeman E. Parathyroid Hormone Deficiency and Excess: Similar Effects on Trabecular Bone but Differing Effects on Cortical Bone. J Clin Endocrinol Metab. 1999; 84(2):718–722. 10022443 10.1210/jcem.84.2.5498

[18] Przedlacki J, Pluskiewicz W, Wieliczko M, et al. Quantitative ultrasound of phalanges and dual-energy X-ray absorptiometry of forearm and hand in patients with end-stage renal failure treated with dialysis. Osteoporos Int. 1999;10(1):1–6. 10501772 10.1007/s001980050186

[19] Abbas SR, Thijssen S, Penne EL, et al. Effect of change in fluid status evaluated by bioimpedance techniques on body composition in hemodialysis patients. J Ren Nutr. 2018;28(3):183–190. 29158062 10.1053/j.jrn.2017.09.002

[20] Katz S, Zlochiver S, Abboud S. Induced current bio-impedance technique for monitoring bone mineral density-a simulation model. Ann Biomed Eng. 2006;34(8):1332–1342. 16807787 10.1007/s10439-006-9146-0

[21] Zhang Z, Yin D, Chen H, et al. Evaluation of anemia, malnutrition, mineral, and bone disorder for maintenance hemodialysis patients based on bioelectrical impedance vector analysis (BIVA). Clin Exp Nephrol. 2020;24(12):1162–1176. 32779058 10.1007/s10157-020-01945-1

[22] Avramovski P, Avramovska M, Lazarevski M, et al. Femoral neck and spine bone mineral density-surrogate marker of aortic calcification in postmenopausal women. Anatol J Cardiol. 2016;16(3):202–209. 26467382 10.5152/akd.2015.6016 PMC5336807

[23] Toussaint ND, Lau KK, Strauss BJ, et al. Determination and validation of aortic calcification measurement from lateral bone densitometry in dialysis patients. Clin J Am Soc Nephrol. 2009;4(1):119–127. 18945998 10.2215/CJN.03410708 PMC2615701

