# Supplementary figures and images for: Value of quantitative ultrasound and bioelectrical impedance analysis in detecting low bone mineral density in hemodialysis
Source: Ren Fail. 2021 Aug 9;43(1):1198–204. doi: 10.1080/0886022X.2021.1959347 (PMC8354166; doi:10.1080/0886022X.2021.1959347)

Supplemental Figures: the scatter plots of QUS and DXA, BIA and DXA

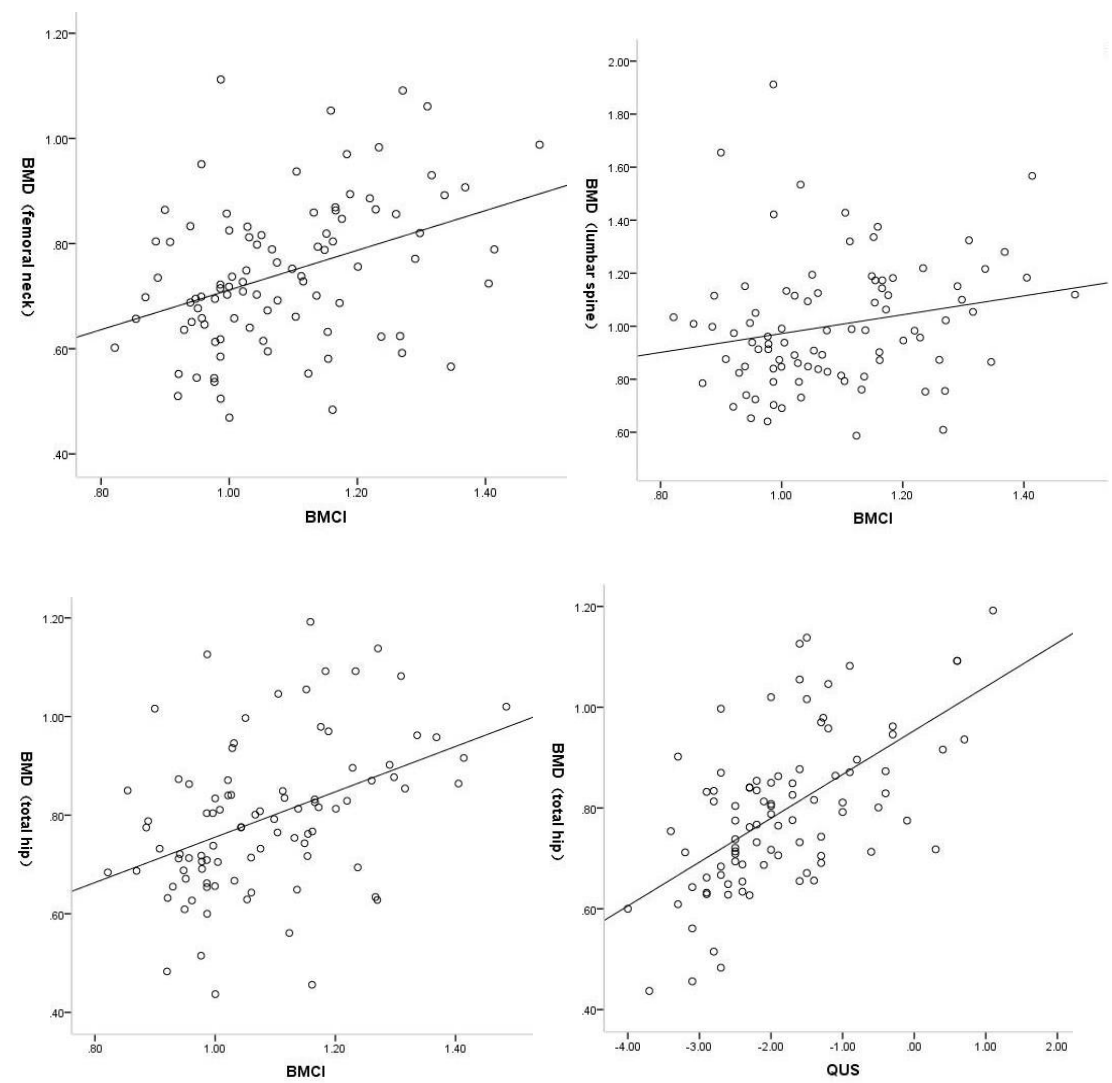

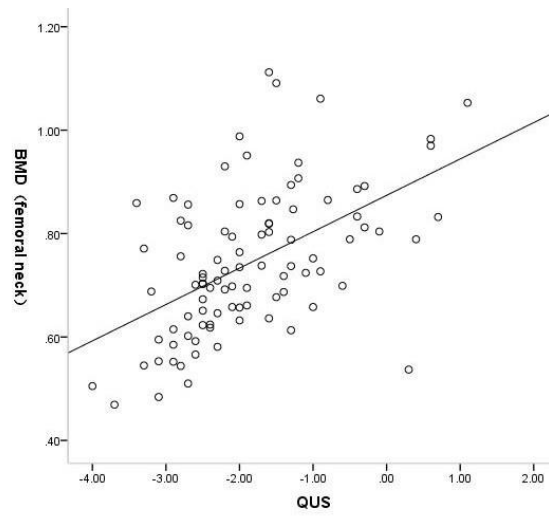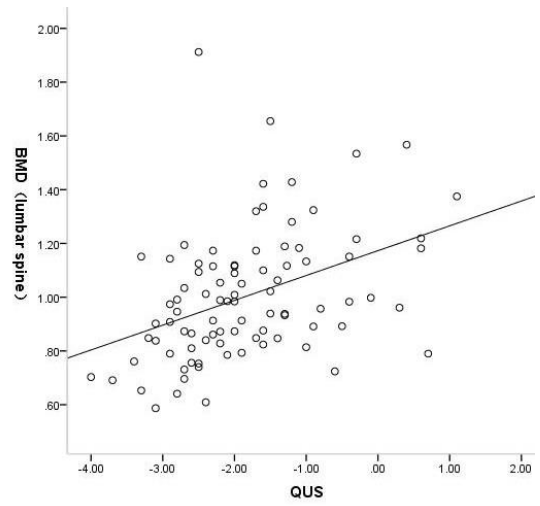

Supplement: Supplemental Material [file IRNF_A_1959347_SM5476.pdf]
